# National Veterans Health Administration MOVE! Weight Management Program Participation During the COVID-19 Pandemic

**DOI:** 10.5888/pcd19.210303

**Published:** 2022-03-10

**Authors:** Kristen E. Gray, Katherine D. Hoerster, Stephanie A. Spohr, Jessica Y. Breland, Susan D. Raffa

**Affiliations:** 1 Health Services Research & Development, VA Puget Sound Health Care System, Seattle Division, Seattle, Washington; 2 Department of Health Systems and Population Health, University of Washington School of Public Health, Seattle, Washington; 3 Mental Health Service, VA Puget Sound Health Care System, Seattle Division, Seattle, Washington; 4 Department of Psychiatry and Behavioral Sciences, University of Washington, Seattle, Washington; 5 National Center for Health Promotion and Disease Prevention, Veterans Health Administration, Durham, North Carolina; 6 Center for Innovation to Implementation, VA Palo Alto Health Care System, Palo Alto, California; 7 Department of Psychiatry & Behavioral Sciences, Duke University School of Medicine, Durham, North Carolina

## Abstract

**Introduction:**

In response to COVID-19, the Veterans Health Administration (VHA) converted appropriate outpatient visits to virtual care, including MOVE! Weight Management Program for Veterans (MOVE!) visits. Before the pandemic, most veterans participated in MOVE! in person, with several telehealth modalities available. We sought to describe national trends in MOVE! participation during the pandemic (March 2020–January 2021) overall and by modality and to compare participation to prepandemic levels.

**Methods:**

We conducted a national retrospective cohort study of veterans who participated in MOVE! from January 2018 through January 2021. We examined MOVE! participation across VHA aggregated at the national level by month, including the number of visits, participants, and new participants in person and via telehealth, including telephone, clinic-to-clinic synchronous video, anywhere-to-anywhere (eg, provider home to patient home) synchronous video, and remote education and monitoring. We also determined the percentage of all MOVE! visits attributable to each modality and the monthly percentage change in participation during the pandemic compared with monthly averages in prior years.

**Results:**

Before March 2020, 20% to 30% of MOVE! was delivered via telehealth, which increased to 90% by April 2020. Early in the pandemic, telephone-delivered MOVE! was the most common modality, but anywhere-to-anywhere synchronous video participation increased over time. Compared with the same months in prior years, total monthly MOVE! participation remained 20% to 40% lower at the end of 2020 and into January 2021.

**Conclusion:**

The VHA MOVE! program rapidly shifted to telehealth delivery of weight management services in response to the pandemic. However, a gap remained in the number of veterans receiving these services compared with prior years, suggesting potential unmet needs for weight management.

**Summary**

**What is already known on this topic?**

The COVID-19 pandemic has affected physical activity and eating behaviors, resulting in weight gain. Health systems may be able to address this problem through behavioral weight management. Little is known about the pandemic’s effects on participation in weight management programs.

**What is added by this report?**

In the Veterans Health Administration, in-person weight management participation declined sharply because of COVID-19, whereas telehealth participation increased, accounting for 90% of participation during the pandemic versus 20% to 30% before. Total participation remained substantially lower into 2021 compared with prior years.

**What are the implications for public health practice?**

The demand for weight management services may increase in the postpandemic recovery period, which could be met by health care systems offering telehealth or hybrid options.

## Introduction

Since 2006, the Veterans Health Administration (VHA) has offered the MOVE! Weight Management Program for Veterans (MOVE!) to veterans receiving care at all facilities to support the more than 80% who have a body mass index (BMI) of 25 or more (1). MOVE! is an evidence-based comprehensive lifestyle intervention combining dietary, physical activity, and behavioral strategies targeting clinically meaningful weight loss (≥5%) delivered in individual or group formats. Patients can participate in person or by using various technologies, including telephone, video conferencing, in-home monitoring with support, and a self-directed mobile app (2,3). MOVE! is effective, resulting in weight loss of 5% or more among 20% to 25% of participants at 12 months (4); telehealth individual modalities have been as effective as in-person group options (5). Before the COVID-19 pandemic, most MOVE! participation was through in-person group sessions at local VHA facilities. In response to the pandemic, on March 15, 2020, VHA leadership directed all facilities to convert in-person care to virtual care where appropriate (6). As of April 2, 2020, in-person MOVE! visits were suspended nationwide and transitioned to telehealth modalities.

Given that higher BMI is associated with increased risk of severe COVID-19 among veterans with a BMI of 30 or more (7), understanding the effect of the pandemic on weight management is critical for anticipating future behavioral weight management needs. This knowledge will help the VHA and other health care systems better understand how to address future local or national disruptions in care. Despite widespread availability of telehealth weight management options in the VHA, little is known about how the COVID-19 pandemic affected trends in MOVE! participation and the uptake of telehealth modalities. With pandemic-associated unintended weight gains and decreases in physical activity (8), there is likely a greater need for weight management services in the postpandemic recovery period, which telehealth may be well-suited to address. This study aims to 1) describe trends in national MOVE! participation overall and by modality and 2) compare participation with prepandemic levels. We hypothesized that in-person program participation would decrease and telehealth participation would increase.

## Methods

We conducted a descriptive retrospective cohort study using VHA clinical and administrative data on MOVE! participation from the VHA Support Service Center MOVE! Visits Report. We examined MOVE! participation aggregated at the national level by month from January 2018 through January 2021. We considered the period of March 2020–January 2021 as “during the pandemic” and before March 2020 as “prepandemic.” This project was considered quality improvement and, per VHA policy, did not require institutional review board approval or waiver. We obtained a nonresearch determination (per VHA Handbook 1058.05) from the VHA National Center for Health Promotion and Disease Prevention.

### MOVE! participation

We examined several dimensions of MOVE! participation each month, including the number of MOVE! visits, the number of MOVE! participants, and the number of new MOVE! participants. We identified MOVE!-related visits by clinic stop codes, which are administrative codes for identifying outpatient care. The combination of up to 2 stop codes indicated the MOVE! visit format (group [373] vs individual [372]) and the delivery modality. The combination of visit format and delivery modality resulted in more than 100 different stop code pairs.

We defined delivery modality as in person, telephone, Clinical Video Telehealth (clinic-to-clinic), VHA Video Connect (anywhere-to-anywhere [eg, provider home to veteran home]), or TeleMOVE! home telehealth. We also created a composite telehealth category, which aggregated telephone, Clinical Video Telehealth, VHA Video Connect, and TeleMOVE! home telehealth. Telephone involves delivery of the standard in-person MOVE! curriculum via telephone. Clinical Video Telehealth and VHA Video Connect are synchronous video sessions. TeleMOVE! home telehealth is an individual remote education and monitoring program completed by veterans at home using an in-home messaging device, browser-based technology, or mobile device and scale. Veterans receive daily education and transmit weight and other relevant health data for monitoring by a care coordinator, who assists participants with behavior change.

The number of MOVE! participants can include veterans more than once across stop codes. If a veteran has a MOVE! visit with a different stop code in the same month, they contribute data to each combination of stop codes (eg, to individual in-person visits and individual telephone visits). Therefore, totals summed across modalities will overestimate the unique number of participants in each month. Veterans are considered new MOVE! participants when they make their first ever MOVE! visit, and they are counted only once for this dimension of MOVE! participation.

### Data analysis

We examined MOVE! participation from January 2018 through January 2021. We first characterized changes over time in the number of visits, participants, and new participants by MOVE! modality (in-person or telehealth), as well as the monthly distribution of MOVE! participation by modality (eg, percentage of all MOVE! visits attributable to each modality). We further examined trends in participation in each telehealth modality by group format versus individual format. To compare MOVE! participation during the COVID-19 pandemic to typical years, we averaged monthly participation in 2018 and 2019, assigning equal weight to each year, and calculated the percentage change in 2020 relative to the prior years’ average for that month. We calculated percentage change in January 2021 relative to the average across January 2019 and 2020, both of which were in the prepandemic period and were most proximal to January 2021 (vs averaging January 2018 and January 2019 for comparisons). We compared data from the same months in prior years because of seasonal variation in MOVE! participation, which precluded comparing data from the height of the pandemic to the months preceding it.

## Results

### Visits

**Figure 1:**
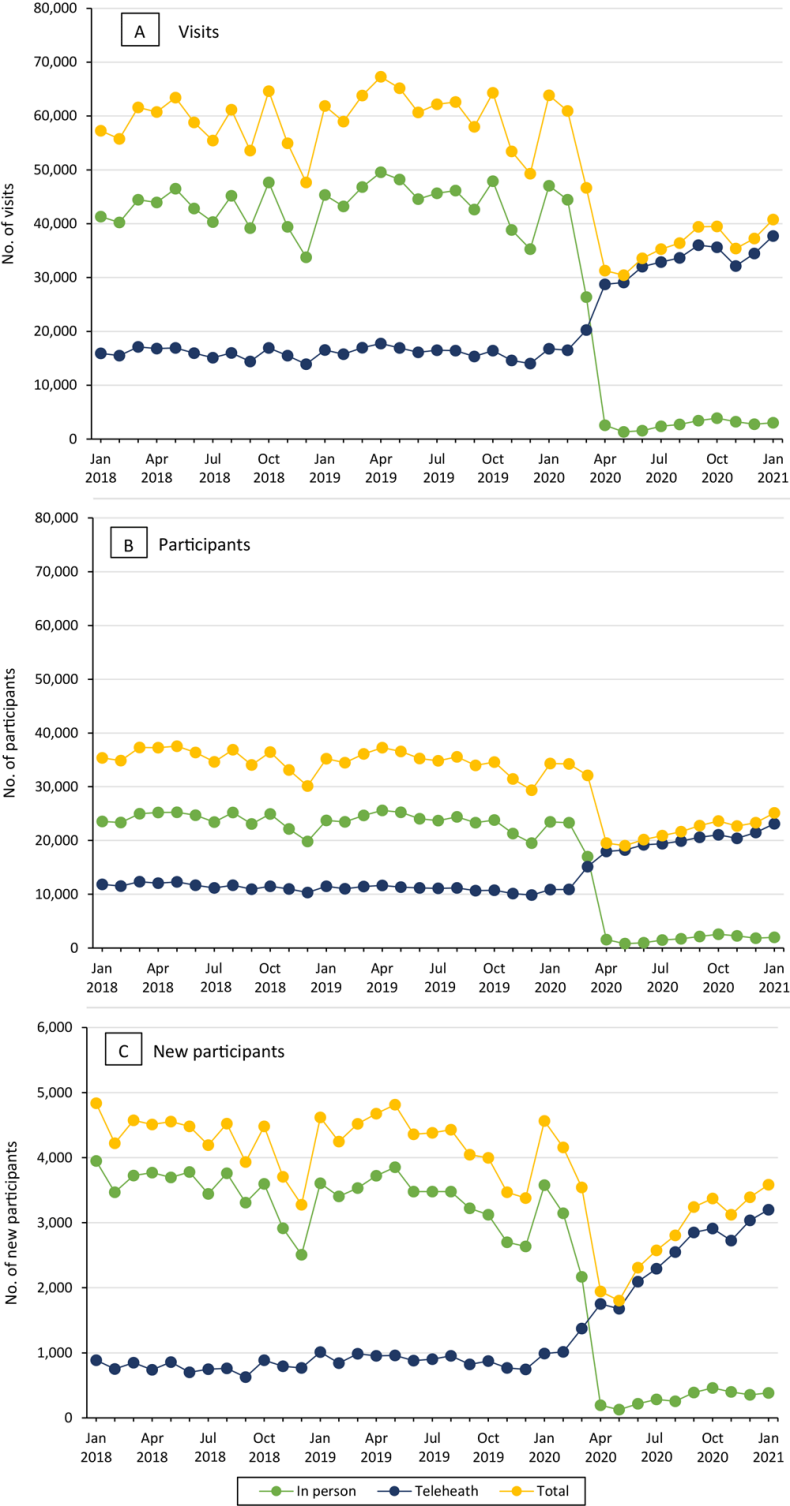
Trends in national Veterans Health Administration MOVE! Weight Management Program participation by modality, January 2018 through January 2021. A, Number of MOVE! visits. B, Number of MOVE! participants. C, Number of new MOVE! participants. Note that the scale in C differs from the scale in A and B.

We found 695,050 MOVE! visits in 2018, 727,585 visits in 2019, and 489,939 visits in 2020. The monthly number of MOVE! visits began to decline sharply in March 2020 because of declines in in-person visits to a nadir in May 2020 of 30,436 visits across modalities (Figure 1). 

**Table:** 

Date	No. of visits			No. of participants			No. of new participants		
	In person	Telehealth	Total	In person	Telehealth	Total	In person	Telehealth	Total
2018-Jan	41,323	15,937	57,260	23,547	11,825	35,372	3,948	887	4,835
2018-Feb	40,245	15,504	55,749	23,337	11,520	34,857	3,468	752	4,220
2018-Mar	44,465	17,139	61,604	24,963	12,357	37,320	3,726	847	4,573
2018-Apr	43,940	16,813	60,753	25,206	12,075	37,281	3,769	740	4,509
2018-May	46,487	16,939	63,426	25,252	12,296	37,548	3,695	858	4,553
2018-Jun	42,833	15,978	58,811	24,694	11,665	36,359	3,779	701	4,480
2018-Jul	40,329	15,106	55,435	23,446	11,178	34,624	3,443	748	4,191
2018-Aug	45,175	16,008	61,183	25,216	11,666	36,882	3,760	763	4,523
2018-Sep	39,182	14,421	53,603	23,088	10,947	34,035	3,308	626	3,934
2018-Oct	47,682	16,936	64,618	24,945	11,499	36,444	3,596	885	4,481
2018-Nov	39,412	15,513	54,925	22,141	10,982	33,123	2,913	793	3,706
2018-Dec	33,782	13,901	47,683	19,838	10,315	30,153	2,508	767	3,275
2019-Jan	45,350	16,539	61,889	23,730	11,492	35,222	3,607	1,010	4,617
2019-Feb	43,204	15,771	58,975	23,466	11,006	34,472	3,405	841	4,246
2019-Mar	46,812	16,984	63,796	24,664	11,454	36,118	3,533	987	4,520
2019-Apr	49,546	17,729	67,275	25,607	11,650	37,257	3,721	955	4,676
2019-May	48,224	16,926	65,150	25,240	11,344	36,584	3,854	959	4,813
2019-Jun	44,562	16,120	60,682	24,061	11,180	35,241	3,479	879	4,358
2019-Jul	45,670	16,505	62,175	23,692	11,118	34,810	3,479	901	4,380
2019-Aug	46,175	16,436	62,611	24,397	11,169	35,566	3,478	953	4,431
2019-Sep	42,625	15,360	57,985	23,302	10,667	33,969	3,223	822	4,045
2019-Oct	47,892	16,419	64,311	23,818	10,757	34,575	3,124	873	3,997
2019-Nov	38,838	14,595	53,433	21,295	10,145	31,440	2,699	768	3,467
2019-Dec	35,267	14,036	49,303	19,527	9,846	29,373	2,636	744	3,380
2020-Jan	47,061	16,768	63,829	23,467	10,851	34,318	3,577	988	4,565
2020-Feb	44,447	16,501	60,948	23,329	10,910	34,239	3,144	1,013	4,157
2020-Mar	26,393	20,250	46,643	17,005	15,114	32,119	2,169	1,372	3,541
2020-Apr	2,557	28,719	31,276	1,546	17,958	19,504	194	1,750	1,944
2020-May	1,334	29,102	30,436	809	18,240	19,049	126	1,677	1,803
2020-Jun	1,571	32,011	33,582	984	19,197	20,181	216	2,094	2,310
2020-Jul	2,381	32,887	35,268	1,503	19,393	20,896	283	2,293	2,576
2020-Aug	2,737	33,660	36,397	1,731	19,914	21,645	255	2,549	2,804
2020-Sep	3,406	36,023	39,429	2,145	20,610	22,755	391	2,851	3,242
2020-Oct	3,879	35,631	39,510	2,571	21,048	23,619	460	2,912	3,372
2020-Nov	3,242	32,141	35,383	2,279	20,412	22,691	399	2,725	3,124
2020-Dec	2,775	34,463	37,238	1,842	21,472	23,314	356	3,035	3,391
2021-Jan	3,038	37,730	40,768	2,000	23,128	25,128	383	3,200	3,583

**Figure 2:**
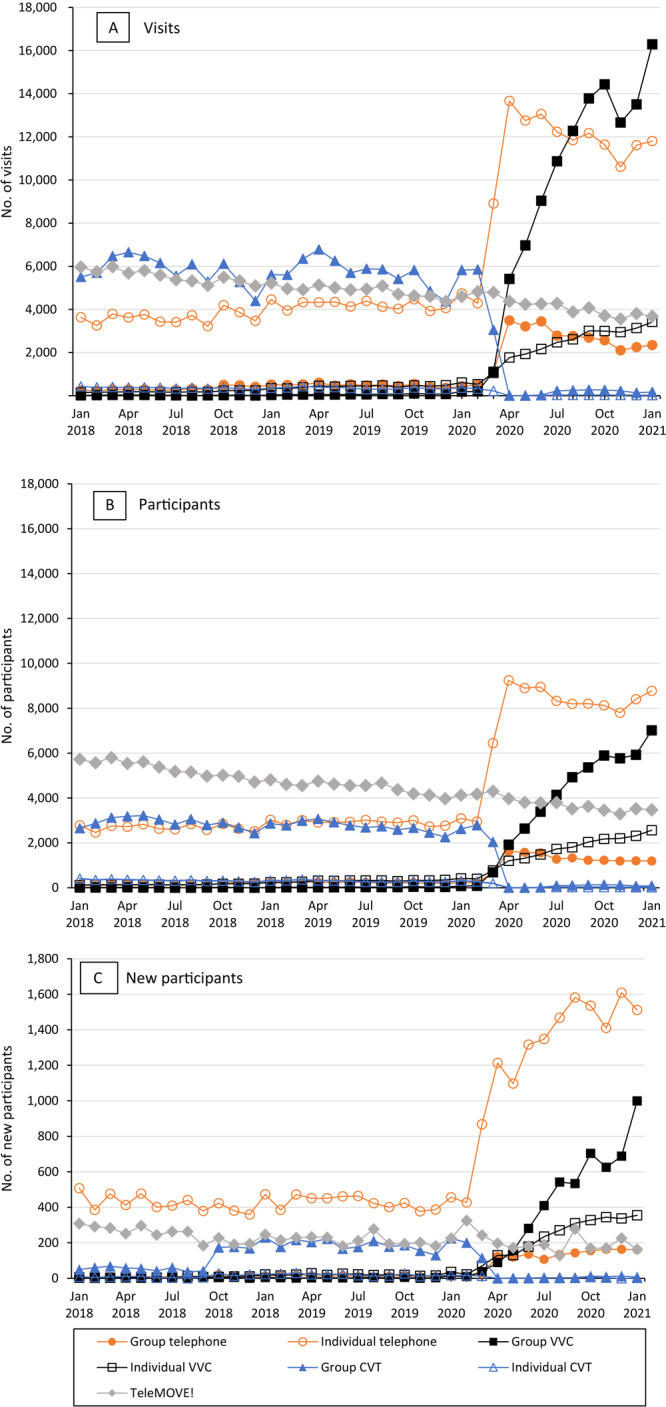
Trends in national Veterans Health Administration telehealth MOVE! Weight Management Program participation by format (group vs individual) and modality, January 2018 through January 2021. A, Number of MOVE! visits. B, Number of MOVE! participants. C, Number of new MOVE! participants. Note that the scale in C differs from the scale in A and B. Abbreviations: VVC, VHA Video Connect; CVT, Clinical Video Telehealth.

We found concomitant increases in telehealth MOVE! modalities, particularly individual telephone visits and group VHA Video Connect visits (Figure 2).

**Table:** 

Date	Group telephone	Individual telephone	Group VHA Video Connect	Individual VHA Video Connect	Group Clinical Video Telehealth	Individual Clinical Video Telehealth	TeleMOVE!
**No. of visits**							
2018-Jan	246	3,630	15	144	5,506	433	5,963
2018-Feb	228	3,252	28	161	5,698	387	5,750
2018-Mar	292	3,785	38	174	6,468	401	5,981
2018-Apr	287	3,621	34	176	6,645	375	5,675
2018-May	295	3,759	36	192	6,475	387	5,795
2018-Jun	249	3,428	23	173	6,150	375	5,580
2018-Jul	263	3,408	16	164	5,556	332	5,367
2018-Aug	317	3,724	9	178	6,094	368	5,318
2018-Sep	276	3,221	19	175	5,295	322	5,113
2018-Oct	497	4,193	28	229	6,120	378	5,491
2018-Nov	478	3,864	24	238	5,262	319	5,328
2018-Dec	410	3,473	6	241	4,390	296	5,085
2019-Jan	511	4,458	30	352	5,605	361	5,222
2019-Feb	502	3,951	49	335	5,597	380	4,957
2019-Mar	524	4,331	47	382	6,344	440	4,916
2019-Apr	604	4,328	53	456	6,774	385	5,129
2019-May	464	4,345	52	419	6,251	391	5,004
2019-Jun	523	4,134	58	452	5,696	351	4,906
2019-Jul	476	4,389	60	449	5,887	304	4,940
2019-Aug	512	4,127	73	473	5,857	307	5,087
2019-Sep	446	4,030	67	391	5,413	307	4,706
2019-Oct	543	4,465	105	475	5,821	376	4,634
2019-Nov	384	3,924	73	437	4,855	315	4,607
2019-Dec	360	4,064	73	484	4,376	317	4,362
2020-Jan	441	4,741	187	608	5,826	366	4,599
2020-Feb	495	4,283	198	535	5,850	322	4,818
2020-Mar	1,118	8,912	1,107	1,055	3,048	225	4,785
2020-Apr	3,484	13,664	5,416	1,769	11	2	4,373
2020-May	3,212	12,754	6,968	1,929	6	2	4,231
2020-Jun	3,448	13,058	9,039	2,161	33	13	4,259
2020-Jul	2,786	12,233	10,865	2,477	221	30	4,275
2020-Aug	2,766	11,846	12,273	2,615	250	30	3,880
2020-Sep	2,688	12,169	13,779	3,005	277	33	4,072
2020-Oct	2,567	11,636	14,433	2,994	267	31	3,703
2020-Nov	2,107	10,605	12,665	2,948	226	37	3,553
2020-Dec	2,248	11,618	13,502	3,131	134	25	3,805
2021-Jan	2,344	11,807	16,290	3,416	169	24	3,680

**No. of participants**							
2018-Jan	127	2,777	9	119	2,655	408	5,730
2018-Feb	108	2,465	14	138	2,873	351	5,571
2018-Mar	131	2,755	15	139	3,128	385	5,804
2018-Apr	129	2,720	17	140	3,180	356	5,533
2018-May	124	2,829	16	141	3,219	352	5,615
2018-Jun	126	2,627	14	143	3,045	330	5,380
2018-Jul	116	2,609	7	131	2,818	316	5,181
2018-Aug	129	2,834	5	144	3,060	339	5,155
2018-Sep	131	2,582	9	148	2,798	301	4,978
2018-Oct	219	2,842	17	178	2,905	320	5,018
2018-Nov	228	2,628	11	175	2,687	285	4,968
2018-Dec	210	2,516	4	187	2,428	261	4,709
2019-Jan	215	3,027	11	244	2,864	319	4,812
2019-Feb	226	2,803	21	246	2,780	325	4,605
2019-Mar	249	3,010	20	266	2,985	371	4,553
2019-Apr	252	2,912	24	317	3,067	326	4,752
2019-May	236	2,924	23	307	2,921	316	4,617
2019-Jun	259	2,933	25	327	2,779	305	4,552
2019-Jul	234	3,015	28	324	2,690	272	4,555
2019-Aug	205	2,941	29	329	2,740	259	4,666
2019-Sep	218	2,900	32	292	2,589	260	4,376
2019-Oct	226	3,001	42	335	2,669	297	4,187
2019-Nov	188	2,730	37	330	2,457	272	4,131
2019-Dec	198	2,772	35	349	2,262	262	3,968
2020-Jan	214	3,094	75	413	2,620	311	4,124
2020-Feb	231	2,952	84	401	2,784	280	4,178
2020-Mar	667	6,443	691	769	2,043	196	4,305
2020-Apr	1,613	9,241	1,920	1,198	7	2	3,977
2020-May	1,585	8,897	2,636	1,317	3	2	3,800
2020-Jun	1,554	8,946	3,390	1,484	22	12	3,789
2020-Jul	1,278	8,328	4,144	1,728	94	28	3,793
2020-Aug	1,332	8,196	4,927	1,807	107	25	3,520
2020-Sep	,1223	8,200	5,365	2,037	126	30	3,629
2020-Oct	1,230	8,130	5,892	2,172	139	26	3,459
2020-Nov	1,194	7,801	5,766	2,203	125	32	3,291
2020-Dec	1,201	8,399	5,928	2,316	82	24	3,522
2021-Jan	1,197	8,770	7,015	2,566	85	22	3,473

**No. of new participants**							
2018-Jan	10	507	0	5	49	8	308
2018-Feb	6	385	2	2	61	5	291
2018-Mar	9	476	0	3	68	8	283
2018-Apr	1	412	1	6	58	10	252
2018-May	11	477	0	7	55	12	296
2018-Jun	8	401	0	4	42	2	244
2018-Jul	8	409	0	3	61	4	263
2018-Aug	9	441	0	13	37	0	263
2018-Sep	6	379	2	7	42	6	184
2018-Oct	22	423	4	7	174	27	228
2018-Nov	14	381	5	10	177	15	191
2018-Dec	13	359	0	10	169	22	194
2019-Jan	11	473	4	22	231	23	246
2019-Feb	19	385	3	16	177	26	215
2019-Mar	18	472	1	21	216	30	229
2019-Apr	14	451	2	28	205	22	233
2019-May	11	451	3	19	222	23	230
2019-Jun	16	461	2	26	167	25	182
2019-Jul	9	464	2	22	176	17	211
2019-Aug	7	423	4	18	211	13	277
2019-Sep	12	401	1	21	178	15	194
2019-Oct	17	424	5	19	187	27	194
2019-Nov	8	377	0	14	156	12	201
2019-Dec	10	387	3	16	130	16	182
2020-Jan	12	456	15	36	226	17	226
2020-Feb	12	427	9	22	202	16	325
2020-Mar	25	868	38	70	115	14	242
2020-Apr	123	1,213	89	129	0	0	196
2020-May	119	1,097	157	130	2	0	172
2020-Jun	136	1,316	281	178	1	1	181
2020-Jul	107	1,348	409	235	4	1	189
2020-Aug	135	1,468	542	270	2	4	128
2020-Sep	143	1,581	533	310	4	2	278
2020-Oct	156	1,536	704	328	13	6	169
2020-Nov	163	1,410	625	345	9	3	170
2020-Dec	163	1,609	688	337	13	0	225
2021-Jan	162	1,512	999	355	7	2	163

**Figure 3:**
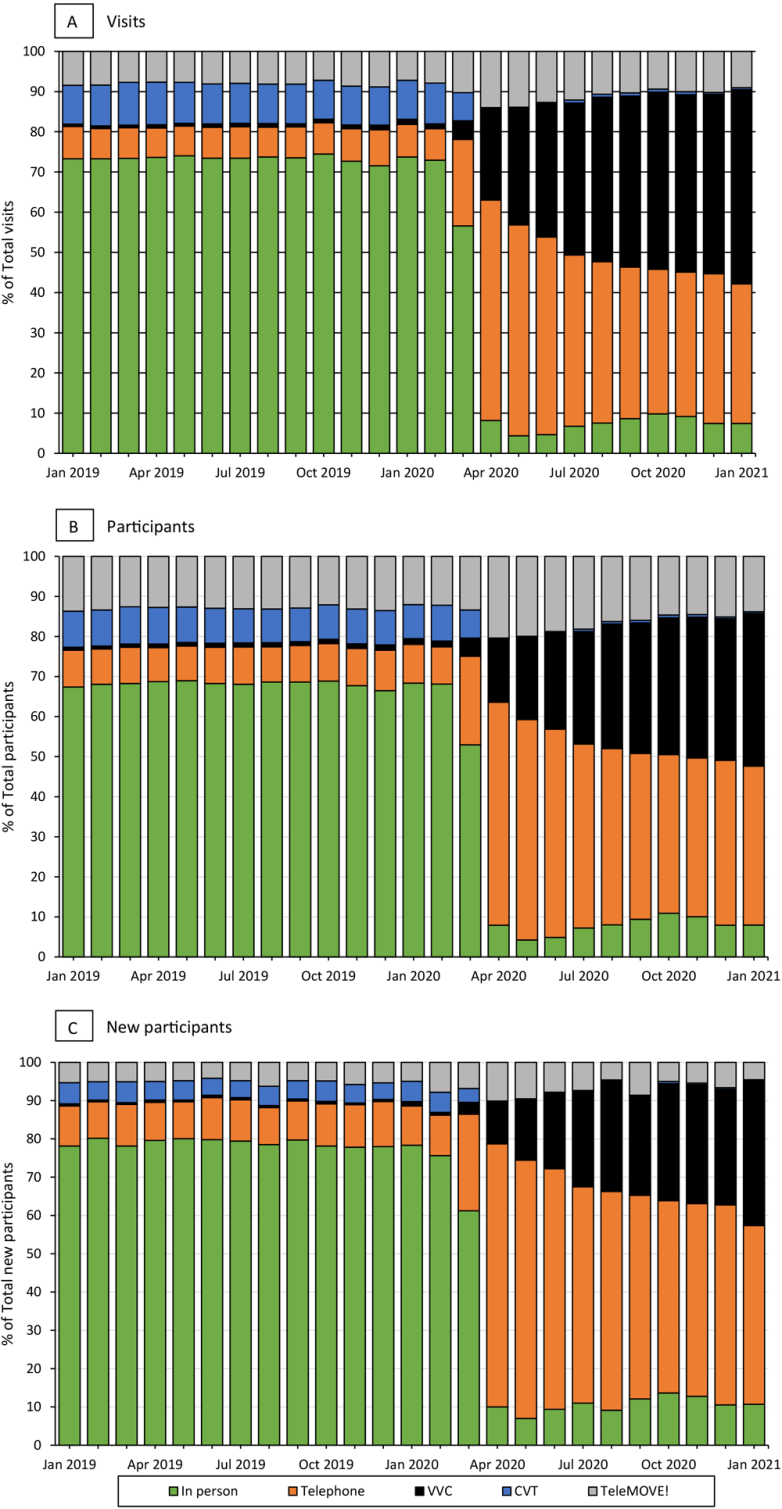
Distribution of national Veterans Health Administration MOVE! Weight Management Program participation by modality, January 2019 through January 2021. A, MOVE! visits. B, MOVE! participants. C, New MOVE! participants. Abbreviations: VVC, VHA Video Connect; CVT, Clinical Video Telehealth.

Before March 2020, telehealth modalities made up approximately 30% of MOVE! visits, which then expanded to 90% or more (Figure 3). In the initial months of the pandemic, telephone visits accounted for the largest proportion of visits, but the proportion declined over time, from 54.8% in April 2020 to 34.7% in January 2021, while the proportion of VHA Video Connect visits increased, from 23.0% in April 2020 to 48.3% in January 2021.

**Table:** 

Date	Percentage of total				
	In person	Telephone	VHA Video Connect	Clinical Video Telehealth	TeleMOVE!
**Visits**					
2019-Jan	73.3	8.0	0.6	9.6	8.4
2019-Feb	73.3	7.6	0.7	10.1	8.4
2019-Mar	73.4	7.6	0.7	10.6	7.7
2019-Apr	73.6	7.3	0.8	10.6	7.6
2019-May	74.0	7.4	0.7	10.2	7.7
2019-Jun	73.4	7.7	0.8	10.0	8.1
2019-Jul	73.5	7.8	0.8	10.0	7.9
2019-Aug	73.7	7.4	0.9	9.8	8.1
2019-Sep	73.5	7.7	0.8	9.9	8.1
2019-Oct	74.5	7.8	0.9	9.6	7.2
2019-Nov	72.7	8.1	1.0	9.7	8.6
2019-Dec	71.5	9.0	1.1	9.5	8.8
2020-Jan	73.7	8.1	1.2	9.7	7.2
2020-Feb	72.9	7.8	1.2	10.1	7.9
2020-Mar	56.6	21.5	4.6	7.0	10.3
2020-Apr	8.2	54.8	23.0	0.0	14.0
2020-May	4.4	52.5	29.2	0.0	13.9
2020-Jun	4.7	49.2	33.4	0.1	12.7
2020-Jul	6.8	42.6	37.8	0.7	12.1
2020-Aug	7.5	40.1	40.9	0.8	10.7
2020-Sep	8.6	37.7	42.6	0.8	10.3
2020-Oct	9.8	35.9	44.1	0.8	9.4
2020-Nov	9.2	35.9	44.1	0.7	10.0
2020-Dec	7.5	37.2	44.7	0.4	10.2
2021-Jan	7.5	34.7	48.3	0.5	9.0

**Participants**					
2019-Jan	67.4	9.2	0.7	9.0	13.7
2019-Feb	68.1	8.8	0.8	9.0	13.4
2019-Mar	68.3	9.0	0.8	9.3	12.6
2019-Apr	68.7	8.5	0.9	9.1	12.8
2019-May	69.0	8.6	0.9	8.8	12.6
2019-Jun	68.3	9.1	1.0	8.8	12.9
2019-Jul	68.1	9.3	1.0	8.5	13.1
2019-Aug	68.6	8.8	1.0	8.4	13.1
2019-Sep	68.6	9.2	1.0	8.4	12.9
2019-Oct	68.9	9.3	1.1	8.6	12.1
2019-Nov	67.7	9.3	1.2	8.7	13.1
2019-Dec	66.5	10.1	1.3	8.6	13.5
2020-Jan	68.4	9.6	1.4	8.5	12.0
2020-Feb	68.1	9.3	1.4	8.9	12.2
2020-Mar	52.9	22.1	4.5	7.0	13.4
2020-Apr	7.9	55.7	16.0	0.0	20.4
2020-May	4.2	55.0	20.8	0.0	19.9
2020-Jun	4.9	52.0	24.2	0.2	18.8
2020-Jul	7.2	46.0	28.1	0.6	18.2
2020-Aug	8.0	44.0	31.1	0.6	16.3
2020-Sep	9.4	41.4	32.5	0.7	15.9
2020-Oct	10.9	39.6	34.1	0.7	14.6
2020-Nov	10.0	39.6	35.1	0.7	14.5
2020-Dec	7.9	41.2	35.4	0.5	15.1
2021-Jan	8.0	39.7	38.1	0.4	13.8

**New participants**					
2019-Jan	78.1	10.5	0.6	5.5	5.3
2019-Feb	80.2	9.5	0.4	4.8	5.1
2019-Mar	78.2	10.8	0.5	5.4	5.1
2019-Apr	79.6	9.9	0.6	4.9	5.0
2019-May	80.1	9.6	0.5	5.1	4.8
2019-Jun	79.8	10.9	0.6	4.4	4.2
2019-Jul	79.4	10.8	0.5	4.4	4.8
2019-Aug	78.5	9.7	0.5	5.1	6.3
2019-Sep	79.7	10.2	0.5	4.8	4.8
2019-Oct	78.2	11.0	0.6	5.4	4.9
2019-Nov	77.8	11.1	0.4	4.8	5.8
2019-Dec	78.0	11.7	0.6	4.3	5.4
2020-Jan	78.4	10.3	1.1	5.3	5.0
2020-Feb	75.6	10.6	0.7	5.2	7.8
2020-Mar	61.3	25.2	3.0	3.6	6.8
2020-Apr	10.0	68.7	11.2	0.0	10.1
2020-May	7.0	67.4	15.9	0.1	9.5
2020-Jun	9.4	62.9	19.9	0.1	7.8
2020-Jul	11.0	56.5	25.0	0.2	7.3
2020-Aug	9.1	57.2	29.0	0.2	4.6
2020-Sep	12.1	53.2	26.0	0.2	8.6
2020-Oct	13.6	50.2	30.6	0.6	5.0
2020-Nov	12.8	50.4	31.0	0.4	5.4
2020-Dec	10.5	52.3	30.2	0.4	6.6
2021-Jan	10.7	46.7	37.8	0.3	4.5

**Figure 4:**
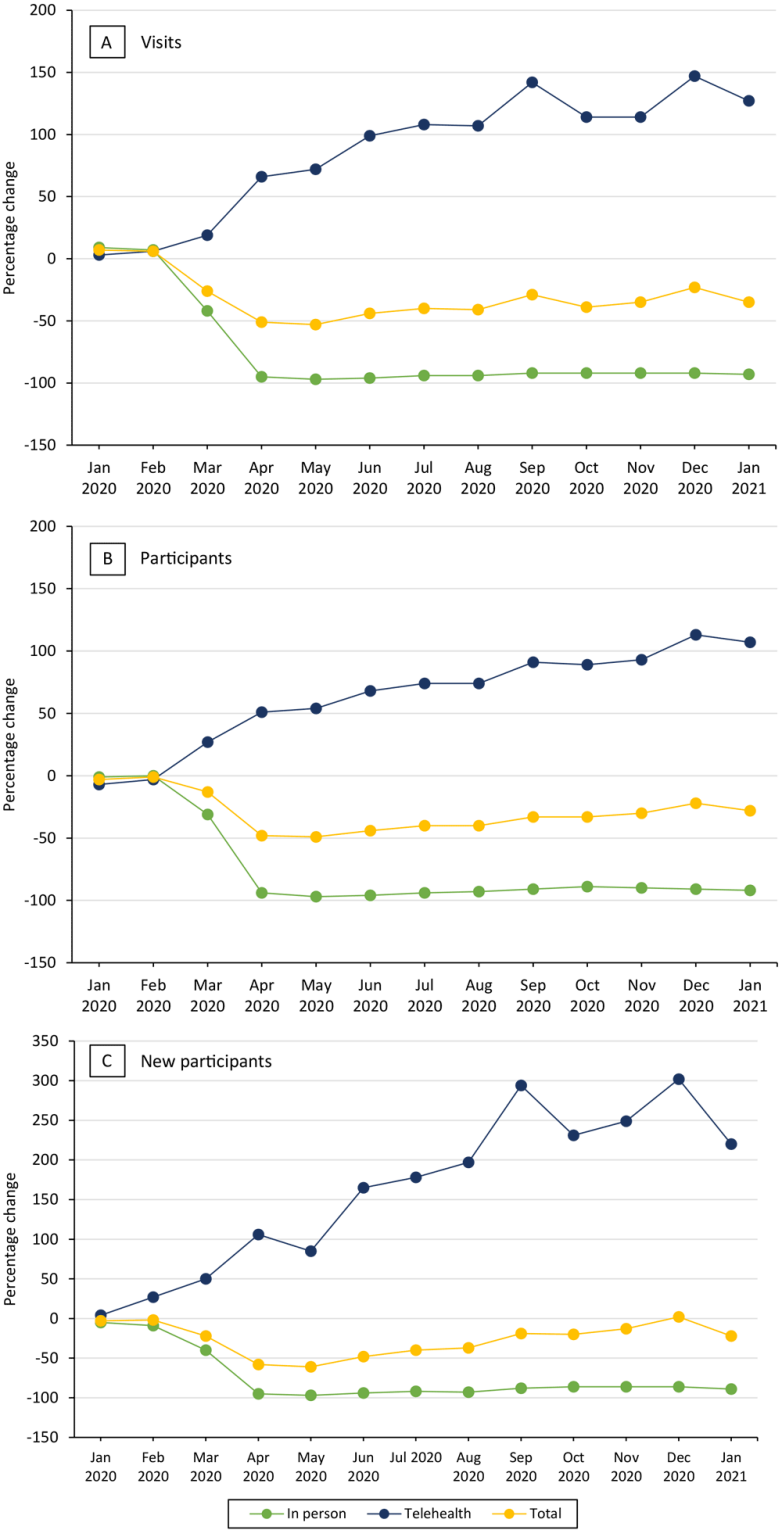
Monthly percentage change in national Veterans Health Administration MOVE! Weight Management Program participation by modality in January 2020 through January 2021 relative to monthly average in prior years. A, MOVE! visits. B, MOVE! participants. C, New MOVE! participants. Note that the scale in C differs from the scale in A and B.

When compared with the monthly average in the years before the pandemic, the number of visits dropped by more than 50% in April 2020, driven by a 95% decline in in-person visits (Figure 4). In subsequent months, in-person visits remained 92% to 97% lower than averages for corresponding months in previous years. In the same period, telehealth visits increased relative to averages in previous years, with percentage increases ranging from 66% in April to 147% in December 2020. This increase mitigated some of the decline in total MOVE! visits compared with prior years, such that differences in total number of visits generally declined over time from 51% lower in April 2020 compared with averages in April 2019 and 2018 to 35% lower in January 2021 compared with January 2020 and 2019.

**Table:** 

Date	Visits, % change			Participants, % change			New participants, % change		
	In person	Telehealth	Total	In person	Telehealth	Total	In person	Telehealth	Total
2020-Jan	9	3	7	−1	−7	−3	−5	4	−3
2020-Feb	7	6	6	0	−3	−1	−9	27	−2
2020-Mar	−42	19	−26	−31	27	−13	−40	50	−22
2020-Apr	−95	66	−51	−94	51	−48	−95	106	−58
2020-May	−97	72	−53	−97	54	−49	−97	85	−61
2020-Jun	−96	99	−44	−96	68	−44	−94	165	−48
2020-Jul	−94	108	−40	−94	74	−40	−92	178	−40
2020-Aug	−94	107	−41	−93	74	−40	−93	197	−37
2020-Sep	−92	142	−29	−91	91	−33	−88	294	−19
2020-Oct	−92	114	−39	−89	89	−33	−86	231	−20
2020-Nov	−92	114	−35	−90	93	−30	−86	249	−13
2020-Dec	−92	147	−23	−91	113	−22	−86	302	2
2021-Jan	−93	127	−35	−92	107	−28	−89	220	−22

### Participants

Trends in the number of participants were similar to trends in the number of visits: declines began in March 2020 for the number of in-person and total participants, with the lowest numbers in May 2020 (Figure 1). The number of telehealth participants increased starting in March 2020 and continued through January 2021, with increases most pronounced in individual telephone participants and group VHA Video Connect participants (Figure 2). Before the pandemic, telehealth accounted for slightly more than 30% of participants in MOVE! but increased to 90% or more after March 2020 (Figure 3). Starting in April 2020, telephone participants accounted for the largest proportion; however, the proportion of VHA Video Connect participants increased over time, from 16% in April 2020 to 38% in January 2021 (Figure 3). Patterns in monthly participants compared with prior years were similar to patterns in visits: total participants declined by nearly 50% for April and May 2020, driven by declines in in-person participants (Figure 4). Compared with averages in prior years, telehealth participants generally increased over time, peaking at 113% in December 2020. In general, deficits compared with previous years became smaller over time, with above-noted 50% fewer total participants in April and May 2020, compared with prior years, down to 28% fewer in January 2021 relative to prior years.

### New participants

The number of new MOVE! participants was 51,280 in 2018, 50,930 in 2019, and 36,829 in 2020. Declines in the number of new participants in telehealth and in-person modalities caused by the pandemic reached lows in May 2020 such that only 1,803 patients began MOVE! in that month compared with 4,813 in May 2019 (Figure 1). Increases in telehealth modalities began in March 2020 and continued through January 2021, with the most notable increases among new participants using individual telephone and group VHA Video Connect (Figure 2). Before the pandemic, telehealth accounted for approximately 20% of new participants and increased to 90% from April 2020 onward (Figure 3). Although telephone was the most common modality for new participants throughout the pandemic (69% in April 2020 and 47% in January 2021 [Figure 3]), the share attributable to VHA Video Connect continued to increase over time, from 11% in April 2020 to 38% in January 2021. When comparing the monthly number of new participants to averages in prior years, the nadir was in May 2020, when the number of new participants was 61% of the average across May 2018 and 2019, attributable to a 97% decline in in-person MOVE! relative to prior years (Figure 4). Starting in March 2020, participants seen via telehealth increased substantially, compared with previous years, and peaked at 302% in December 2020. Declines in monthly new participants compared with prior years decreased over time, such that in December 2020, there was a slight increase in new participants relative to December of prior years (2%). However, in January 2021 this dipped back to 22% lower than previous years.

## Discussion

We examined national trends in VHA MOVE! Weight Management Program participation before and during the COVID-19 pandemic, from January 2018 through January 2021. As anticipated, we found dramatic declines in MOVE! participation overall, particularly for in-person modalities, beginning in March 2020. This coincided with general national VHA guidance in mid-March 2020 to convert appropriate outpatient appointments to virtual modalities (6) and subsequent MOVE!-specific national guidance to suspend in-person MOVE! visits for at least 30 days. The MOVE! program and providers responded rapidly, with 90% of participation transitioning to telehealth modalities by April 2020. In the initial months of the pandemic, telephone modalities accounted for the greatest proportion of MOVE! participation, but the share of anywhere-to-anywhere synchronous video (VHA Video Connect) participation increased over time. Compared with the same month in prior years, total monthly MOVE! participation (in person and telehealth) was 50% to 60% lower in April and May 2020. After May 2020, deficits relative to prior years generally became smaller over time (eg, approximately 40% lower in July and August 2020). However, MOVE! participation remained 20% to 30% lower in the latter months of 2020 and into January of 2021 compared with the same months in prior years.

The increase in VHA Video Connect participation over time was likely influenced by efforts to educate providers and patients on this newer technology as well as infrastructure improvements to increase bandwidth available for video appointments (6). The high utilization of telephone visits, particularly early in the pandemic, may reflect initial national guidance to use the modality with the lowest technology requirement (such as secure messaging or telephone) unless video visits had been established for MOVE! or a veteran preferred a video visit. Home telehealth (ie, TeleMOVE!) remained relatively stable over time. This stability was likely related to new COVID-19 home telehealth monitoring protocols that were developed and guidance to facilities to consider COVID-19 monitoring needs before enrolling new TeleMOVE! participants. At facilities where clinician resources were limited (eg, MOVE! staff reassigned to provide COVID-19 care), MOVE! teams were encouraged to prioritize telehealth options according to patient need and local context.

Our findings highlight that, although the VHA had existing telehealth infrastructure and programming, MOVE! participation in 2020 was 30% lower than in previous years. Therefore, many patients who would have otherwise received weight management services did not because of the pandemic. Given COVID-19–related declines in physical activity, increases in sedentary behaviors, and changes in eating patterns, there is a pressing need for accessible weight management services now and in the future (9,10). Contributors to changes in health behaviors may include COVID-19 restrictions leading to shifts in routine or access to healthy foods and safe spaces for physical activity (eg, lack of access to gyms or exercise partners). Psychological distress also increased markedly during the pandemic, which may be a factor in worsening activity and eating behaviors, given that conditions such as depression and posttraumatic stress disorder affect health behaviors and weight (10–13). Pandemic-associated stress may also lead patients to prioritize other aspects of their health (eg, coping with distress by eating comfort foods) and lives (eg, virtual schooling of children) over weight management. Related to these and other factors, 42% of US adults reported gaining unwanted weight during the pandemic, with an average gain of 29 pounds (8). With high rates of mental health conditions, food insecurity, and limited social support, veterans may be especially vulnerable to COVID-19–related behavior change and weight gain (14–18). Weight management programs, including MOVE!, may need to incorporate additional strategies to help participants overcome the challenges to modifying health behaviors in the context of heightened pandemic-related stress and anxiety (19).

Our findings also suggest that barriers to telehealth may remain at the patient, provider, or health care system level. One such potential barrier is the lack of technology necessary for telehealth weight management services. The most common telehealth MOVE! modality early in the pandemic was telephone, but use of anywhere-to-anywhere synchronous video increased over time. Veteran preferences for MOVE! participation format and modality during and after the pandemic have not been broadly assessed. However, a survey among 58 veterans who were seen during the pandemic in an intensive weight management clinic that provides interdisciplinary pre- and postbariatric surgery care may provide some insights (20). In that study, half of participants indicated telephone visits were as good as in-person visits, and a similar proportion said they would prefer to have all visits over the telephone even after pandemic-related restrictions to in-person visits were lifted (20). Although participants were not offered video visits, they were queried about preferences for video. Nearly half indicated they would have preferred video to telephone visits if they were available, and 37% endorsed a preference for all video visits after restrictions were lifted (20). A substantial number of veterans lack the technology needed for video visits, including compatible devices or high-speed internet (21). These barriers differentially affect older, unhoused, and rural veterans (22). To address these gaps, the VHA implemented the Digital Divide Consult, which enables patients to obtain internet services or technology needed for VHA telehealth and provides robust technology support and digital skills classes (23). However, the VHA will need to evaluate the extent to which the pandemic exacerbated disparities in access and the success of the Digital Divide Consult in addressing these disparities. Given anticipated long-term shifts toward reliance on telehealth, the VHA must continue to ensure equitable access for all veterans.

Strategies to promote weight management access for VHA patients may include prioritizing a return to face-to-face modalities as soon as it is safe to do so and ensuring safe practices for face-to-face visits, especially group programs, in accordance with local and federal guidance, including use of face masks, social distancing, proper ventilation, hand hygiene, and encouraging vaccination and boosters. Other strategies could include offering hybrid models with in-person and telehealth options; continuing to provide MOVE! via synchronous video and telephone; and expanding self-directed options, like app, online, or DVD-based programs, which have been effective in non-VHA settings and are currently being tested in the VHA (24–26). Offering telehealth or hybrid options may also enhance access to weight management services in non-VHA settings, where telehealth modalities may be less robust than in the VHA. For example, of the nearly 2,000 Centers for Disease Control and Prevention–recognized Diabetes Prevention Programs, which lead to clinically significant weight loss (27), less than one-quarter have telehealth options (28). Health care systems can also consider delivering weight management programs — in person, telehealth, or hybrid — in nontraditional settings, such as within mental health care, to promote access among specific patient populations (29).

Our study has several important strengths and limitations. We had readily available data on a nationally implemented weight management program with hundreds of thousands of visits over several years, allowing for investigation of trends over time. We also had information on types of program participation, including several different telehealth options, which facilitated examination of uptake of individual telehealth modalities before and during the pandemic. Limitations include use of data aggregated at the national level, precluding examination of the influence of patient-, facility-, or regional-level characteristics on MOVE! participation or understanding variation in the effects of COVID-19 on MOVE! participation. Furthermore, our study did not allow for evaluation of patient attitudes about access and preferred modalities, barriers to service delivery, or patient outreach during the pandemic. Future work should seek to understand patient perspectives on weight management program participation during COVID-19 and evaluate facility- and regional-level differences in the effects of COVID-19 on participation. We also were not able to examine trends in self-directed MOVE! participation (eg, the MOVE! app), although there is insufficient evidence to determine whether self-directed weight management programs without a clinical component are as effective as clinical interventions (30). Our findings reflect data from a single large integrated health care system and may not be generalizable to other health care systems or weight management programs, particularly those that did not offer telehealth options before the pandemic. Lastly, information on one dimension of participation — the number of monthly MOVE! participants — was limited by the potential for participants to be counted more than once in a given month if they participated in 2 or more MOVE! modalities or formats. Because of the transition of MOVE! to telehealth in March 2020, patients were more likely to be duplicated in the early months of the pandemic, because they were more likely to participate in more than 1 modality. Relatedly, we could not calculate yearly totals for participants, because individuals would contribute data to each month in which they participated in MOVE!, leading to substantial overestimates of the total annual number of participants.

In this study, we found a rapid transition of the VHA MOVE! Weight Management Program to telehealth modalities at the height of the COVID-19 pandemic. Although the VHA had been delivering MOVE! via telehealth before the pandemic, we found a sizable gap in total MOVE! participation compared with prior years that persisted into January 2021. Findings suggest potential unmet needs for weight management now and into the future, some of which telehealth may be able to address. Additional research is needed to understand whether these gaps continue and the factors driving them (eg, patient-, facility-, and regional-level factors) to promote equitable access to weight management services.
